# Relationships between dental hygienists’ work environment and patient safety culture

**DOI:** 10.1186/s12913-019-4136-8

**Published:** 2019-05-10

**Authors:** Eun-Mi Choi, So-Jung Mun, Won-Gyun Chung, Hie-Jin Noh

**Affiliations:** 1 Department of Dental Hygiene, Graduate School, Yonsei University, Seoul, Republic of Korea; 2 Department of Dental Hygiene, Yonsei University Wonju College of Medicine, 20 Ilsanro, Wonju, Kangwondo 26426 Republic of Korea

**Keywords:** Dental hygienist, Patient safety culture, Work environment

## Abstract

**Background:**

Patient safety culture is a core factor in increasing patient safety, is related to the quality of medical service, and can lower the risk of patient safety accidents. However, in dentistry, research has previously focused mostly on reporting of patient safety accidents. Dental professionals’ patient safety culture must therefore first be assessed, and related factors analyzed to improve patient safety.

**Methods:**

This cross-sectional study completed a survey on 377 dental hygienists working in dental settings. To assess patient safety culture, we used a survey with proven validity and reliability by translating the Hospital Survey on Patient Safety Culture (HSOPS) developed by Agency for Healthcare Research and Quality (AHRQ) into Korean. Response options on all of the items were on 5-point Likert-type scales. SPSS v21 was used for statistical analysis. The relationships between workplace factors and patient safety culture were examined using t-tests and one-way analysis of variance (ANOVA) tests(*p* < 0.05).

**Results:**

The work environment of dental hygienists has a close relationship with patient safety. Dental hygienists working ≥40 h/week in Korea had a significantly lower for patient safety grade than those working < 40 h/week. When the number of patients per day was less than 8, the safety level of patients was significantly higher. And significant differences were found depending on institution type, institution size.

**Conclusions:**

In order to establish high-quality care and patient safety system practical policies must be enacted. In particular, assurance in the quality of work environment such as sufficient staffing, appropriate work hours, and enough rest must first be realized before patient safety culture can easily be formed.

## Background

Managing the quality of healthcare comprises individual and systematic efforts by healthcare personnel and facilities to ensure that patients safely receive quality medical services [1]. Patients’ safety-related accidents at healthcare facilities range from minor problems to permanent damage or death. The Institute of Medicine reported that deaths caused by preventable medical errors at healthcare facilities ranged between 44,000 and 98,000 per year in the United States [2]. The Korea Centers for Disease Control and Prevention estimated about a 9.2% likelihood of medical accidents among hospital inpatients, a 7.4% likelihood of death due to medical errors, and a 43.5% likelihood of prevention, suggesting a need for accident prevention [3].

Entities that evaluate healthcare facilities perform quality assessments that include criteria on patient safety [4–7]. The Joint Commission on Accreditation of Healthcare Organization (JCAHO) in the United States has included patient safety in its evaluation standards since 2003 [5]. In South Korea, patient safety has been a healthcare facility assessment criterion since 2004, and it gradually has become an important evaluation goal [6, 7]. The Korean Patient Safety Act mandates specific rules regarding aspects of prevention, such as the scope of patients’ safety-related accidents, reporting, and education to create a culture that encourages activities for improving patient safety [4].

One basic principle of patient safety is patient safety culture, which refers to ranking patient safety as the highest priority during all healthcare and medical procedures at all healthcare facilities [8]. Research on this culture is consistently increasing [9–14]. Some studies suggest significant relationships exist between the extent of a patient safety culture, number of in-hospital deaths, medication administration errors, and rehospitalizations, and that a patient safety culture positively relates to satisfaction among patients and their families [10–14].

Dental services typically are performed on an outpatient basis, but many dental procedures use potentially dangerous drugs and complex equipment. Various health risks accompany many of the frequently performed surgical treatments, such as cross-contaminations that threaten the patients and their healthcare providers [15]. Adverse dental outcomes include pain, infection, hard tissue damage, and nerve injury. About 88% of dental procedures were found to cause temporary, moderate, or severe harm to the patient [16].

Although the need for research on patient safety cultures to lower the risk of accidents and potential risk factors is emphasized in medicine [9], previous research on patient safety cultures in dentistry mostly have focused on reporting patients’ safety problems [17–23]. A patient safety culture is vital to increasing patient safety, it relates to the quality of medical services, and it might lower the risks of accidents [10–14]. Therefore, dental professionals’ patient safety cultures should be assessed and factors expected to improve patient safety should be analyzed. This study assessed the patient safety cultures in a sample of dental hygienists with various dental jobs, such as receptionist, preventive dentistry, oral health educator and/or counselor, healthcare collaborator, and hospital administrator at dental healthcare facilities. Additionally, it explored the relationship between the workplace environment and the patient safety culture.

## Methods

### Participants

A convenience sample of dental hygienists at dental clinics or hospitals was obtained through online recruiting. Between March 2 and March 27 of 2015, 462 completed questionnaires were obtained. Questionnaires with incomplete or unusable answers were dropped, totaling 377 dental hygienists in the final analyses. G-power 3.1 was used to calculate the appropriate sample size of 280 to analyze four descriptive variables, a statistical significance cutoff level (α) of 0.05, power (1-β) of 95%, and effect size of 0.25. Approval of this study was obtained from the research ethics committee at Y University (Approval number: YWDR-15-2-006).

### Survey instrument

#### Patient safety culture

The questions used to assess patient safety culture had proven validity and reliability [24]. The Hospital Survey on Patient Safety Culture (HSOPS) developed by the Agency for Healthcare Research and Quality (AHRQ) was translated into Korean [25]. The 38 items covered 10 aspects of patient safety culture: (1) eight questions on patient safety policies across hospital units, (2) five questions on feedback and openness of communication about patient safety, (3) three questions on supervisor/manager democratic expectation/actions, (4) three questions on frequency of reported incidences, (5) four questions on within-unit teamwork for patient safety, (6) six questions on systems and procedures for patient safety, (7) three questions on strict manager responses to errors, (8) two questions on concerns about errors, (9) two questions on organizational training and responses, and (10) two questions on workload. Response options on all of the items were on five-point Likert-type scales, where 1 = *strongly disagree*, 2 = *disagree*, 3 = *neither agree nor disagree*, 4 = *agree*, and 5 = *strongly agree*.

#### Workplace environment

The workplace environment covered work experience, number of hours worked per week, facility type, facility size, number of patients per dental hygienist per day, and certification evaluation of the facility. A previous study in Korea on adherence to safety procedures by work experience found less than 50% adherence among workers with 1 to 2 years of clinical experience, 50–60% adherence among those with three through 8 years of experience, and 60% adherence among those with nine or more years of experience [26]. Therefore, the variable measuring work experience was categorized as 1 to 2 years, three through 8 years, and nine or more years for the analyses [26].

The number of hours worked per week was grouped into less than 40 h and 40 or more hours based on South Korea’s legal standard of a 40-h workweek [27]. The type of dental facility was either a clinic-level or a hospital-level medical facility based on the specifications in South Korea’s healthcare laws [28]. Clinic-level facilities were dental clinics and networked dental clinics, and hospital-level facilities were dental hospitals and general hospitals. The size of the facility was measured by the number of dental chairs and categorized into four groups. Based on the 2016 South Korean Patient Investigation Report [29] results of 7.5 patients per dental hygienist per day on average, the average number of patients per day was divided into two groups (less than eight and eight or more). Facility certification classification was based on whether the facility was certified by the Ministry of Health and Welfare or JCAHO.

### Statistical analysis

First, descriptive statistics were used to summarize the variables in the analysis. The relationships between workplace factors and patient safety culture were examined using *t*-tests and one-way analysis of variance (ANOVA) tests. Cronbach’s alpha reliability statistics on the 38 questions on patient safety culture ranged from 0.641–0.882. Two items from the organizational training and response group and two workload items were omitted because their Cronbach’s alpha values were less than 0.6. The final analysis included 34 items in eight areas. PASW statistics 21.0 was used to perform the statistical analysis and statistical significance was set at 0.05.

## Results

### Patient safety culture

About 50.4% of the respondents reported that their workplace had a patient safety culture. Regarding the components of patient safety, “policy across hospital units” was the highest (65.1%), followed by within-unit teamwork for patient safety (64.0%) and supervisor/manager democratic expectation/actions (59.6%). Concerns about errors (39.4%) and frequency of reported incidences (21.6%) were the lowest (Table 1).

**Table 1:** Average percent positive dimension score of all respondents(*N* = 377)

Dimension	Positive response rate	Mean ± SD
A. Patient safety policy across hospital units (Cronbach’s α = 0.77)	65.1%	3.69 ± 0.54
1–1 Things “fall between the cracks” when transferring patients from one unit to another^+^	70.3%	3.78 ± 0.79
1–2 Important patient care information is often lost during shift changes^+^	77.7%	3.90 ± 0.78
1–3 Problems often occur in exchange of information across hospital units^+^	55.4	3.56 ± 0.87
1–4 Hospital units do not coordinate well with each other^+^	70.0%	3.79 ± 0.79
1–5 There is good cooperation among hospital units that need to work together	56.2%	3.52 ± 0.94
1–6 It is often unpleasant to work with staff from other hospital units^+^	68.4%	3.77 ± 0.90
1–7 Hospital units work well together to provide the best care for patients	65.8%	3.73 ± 0.88
1–8 Hospital management seems interested in patient safety only after an adverse event happens^+^	56.8%	3.51 ± 1.01
B. Feedback and openness of communication for patient safety (Cronbach’s α = 0.81)	55.7%	3.55 ± 0.61
2–1 We are given feedback about changes put into place based on event reports	49.3%	3.47 ± 0.84
2–2 We are informed about errors that happen in this unit	35.3%	3.18 ± 0.84
2–3 In this unit, we discuss ways to prevent errors from happening again	45.9%	3.35 ± 0.85
2–4 Staff will freely speak up if they see something that may negatively affect patient care	75.9%	3.87 ± 0.73
2–5 Staff feel free to question the decisions or actions of those with more authority	72.1%	3.88 ± 0.80
C. Supervisor /manager democratic expectation/actions (Cronbach’s α = 0.62)	59.6%	3.58 ± 0.63
3–1 My supervisor/manager says a good word when he/she sees a job done according to established patient safety procedures	40.8%	3.22 ± 0.89
3–2 My supervisor/manager seriously considers staff suggestions for improving patient safety	63.7%	3.60 ± 0.79
3–3 My supervisor/manager overlooks patient safety problems that happen over and over^+^	74.3%	3.91 ± 0.84
D. Frequency of events reported (Cronbach’s α = 0.88)	21.6%	2.68 ± 0.94
4–1 When a mistake is made, but is caught and corrected before affecting the patient, how often is this reported?	29.4%	2.92 ± 1.04
4–2 When a mistake is made, but has no potential to harm the patient, how often is this reported?	18.6%	2.56 ± 1.06
4–3 When a mistake is made that could harm the patient, but does not, how often is this reported?	16.7%	2.55 ± 1.03
E. Teamwork within units for patient safety (Cronbach’s α = 0.75)	64%	3.65 ± 0.61
5–1 People support one another in this unit	73.5%	3.83 ± 0.67
5–2 When a lot of work needs to be done quickly, we work together as a team to gets the work done	77.2%	3.90 ± 0.73
5–3 In this unit, people treat each other with respect	72.1%	3.84 ± 0.79
5–4 We have enough staff to handle the workload	33.2%	3.04 ± 0.98
F. System and procedure for patient safety (Cronbach’s α = 0.69)	43.6%	3.28 ± 0.59
6–1 We are actively doing things to improve patient safety	58.1%	3.58 ± 0.82
6–2 After we make changes to improve patient safety, we evaluate their effectiveness	41.6%	3.22 ± 0.99
6–3 When an event is reported, it feels like the person in being written up, not the problem^+^	39.5%	3.18 ± 0.94
6–4 We work in “crisis mode” trying to do too much, too quickly^+^	33.2%	3.04 ± 0.97
6–5 Our procedures and systems are good at preventing errors from happening	44.6%	3.34 ± 0.81
6–6 Staff in this unit work longer hours than is best for patient care^+^	44.8%	3.30 ± 1.13
G. Strict manager response to error (Cronbach’s α = 0.64)	54.3%	3.50 ± 0.70
7–1 Staff are afraid to ask question the when something does not seem right^+^	49.9%	3.40 ± 0.99
7–2 Hospital management provides a work climate that promotes patient safety	55.2%	3.51 ± 0.84
7–3 The actions of hospital management show that patient safety is a top priority	57.8%	3.59 ± 0.92
H. Concern for error (Cronbach’s α = 0.85)	39.4%	3.20 ± 1.03
8–1 Staff feel like their mistakes are held against them^+^	44.0%	3.31 ± 1.10
8–2 Staff worry that mistakes they make are kept in their personnel file^+^	34.7%	3.10 ± 1.11

* by Descriptive Statistics, ^+^inverse coding

### The relationship of workplace environment to patient safety culture

Table 2 presents the statistics about the workplace environment and its relationship to a patient safety culture. About 62.3% of the 377 respondents reported working more than 40 h per week, the respondents were more likely to work at clinic-level (56.2%) than hospital-level (43.8%) facilities, and they were more likely to treat more than eight than to treat eight or less patients per day (66.6%). Statistically significant differences were found regarding number of hours worked per week, facility type, facility size, and number of patients treated per day (*p* < 0.05).

**Table 2:** The relationship between Work Environment and patient safety culture(*N* = 377)

Work Environment	Category	N	Overall	A	B	C	D	E	F	G	H
				Mean ± SD	Mean ± SD	Mean ± SD	Mean ± SD	Mean ± SD	Mean ± SD	Mean ± SD	Mean ± SD
Working experience^‡^	1–2 year^a^	152	3.39 ± 0.03	3.77 ± 0.05	3.50 ± 0.05	3.59 ± 0.05	2.66 ± 0.07	3.63 ± 0.05	3.32 ± 0.05	3.52 ± 0.06	3.16 ± 0.08
	3–8 year^b^	156	3.39 ± 0.03	3.63 ± 0.04	3.60 ± 0.05	3.63 ± 0.05	2.69 ± 0.08	3.67 ± 0.04	3.23 ± 0.05	3.50 ± 0.05	3.18 ± 0.08
	≥9 year^c^	69	3.39 ± 0.05	3.67 ± 0.06	3.55 ± 0.07	3.44 ± 0.07	2.68 ± 0.11	3.68 ± 0.07	3.29 ± 0.06	3.46 ± 0.09	3.37 ± 0.13
	*p*-value		0.99	0.08	0.41	0.13	0.96	0.834	0.45	0.81	0.35
	Scheffe										
Working hours per week^†^	≤ 40 h	142	3.45 ± 0.39	3.78 ± 0.52	3.58 ± 0.65	3.62 ± 0.61	2.82 ± 0.90	3.69 ± 0.59	3.16 ± 0.66	3.57 ± 0.72	3.37 ± 1.03
	> 40 h	235	3.36 ± 0.40	3.64 ± 0.55	3.53 ± 0.59	3.56 ± 0.65	2.59 ± 0.95	3.63 ± 0.62	3.35 ± 0.54	3.46 ± 0.68	3.11 ± 1.01
	*p*-value		0.03^*^	0.01^*^	0.43	0.32	0.02^*^	0.34	< 0.001^*^	0.14	0.02^*^
Dental institution type^†^	Clinic-level	212	3.43 ± 0.02	3.68 ± 0.04	3.58 ± 0.04	3.61 ± 0.04	2.81 ± 0.06	3.70 ± 0.04	3.23 ± 0.04	3.42 ± 0.05	3.42 ± 0.07
	Hospital-level	165	3.34 ± 0.04	3.71 ± 0.04	3.52 ± 0.04	3.54 ± 0.05	2.51 ± 0.07	3.60 ± 0.05	3.34 ± 0.05	3.61 ± 0.05	2.93 ± 0.07
	*p*-value		0.033^*^	0.56	0.35	0.256	< 0.001^*^	0.13	0.09	0.01^*^	0.001^*^
Number of unit chair^‡^	≤ 7^a^	108	3.48 ± 0.04	3.78 ± 0.05	3.69 ± 0.06	3.74 ± 0.06	2.83 ± 0.09	3.78 ± 0.05	3.11 ± 0.06	3.49 ± 0.06	3.40 ± 0.10
	8-13^b^	90	3.31 ± 0.04	3.58 ± 0.05	3.38 ± 0.07	3.44 ± 0.07	2.53 ± 0.09	3.56 ± 0.06	3.19 ± 0.07	3.45 ± 0.07	3.38 ± 0.11
	14-25^c^	89	3.32 ± 0.04	3.59 ± 0.05	3.50 ± 0.06	3.51 ± 0.07	2.79 ± 0.10	3.49 ± 0.07	3.32 ± 0.04	3.32 ± 0.08	3.08 ± 0.09
	≥ 26^d^	90	3.44 ± 0.05	3.80 ± 0.06	3.60 ± 0.06	3.59 ± 0.06	2.53 ± 0.11	3.76 ± 0.07	3.53 ± 0.06	3.76 ± 0.07	2.93 ± 0.11
	*p*-value		0.01^*^	< 0.001^*^	< 0.001^*^	0.01^*^	0.03^*^	< 0.001^*^	< 0.001^*^	< 0.001^*^	< 0.001^*^
	Scheffe		a > b		a > b	a > b		a > c	a,b < d	b,c < d	d < a,b
Number of patients per day per dental hygienist^†^	≤8 Patients	126	3.48 ± 0.04	3.85 ± 0.05	3.71 ± 0.05	3.70 ± 0.06	2.58 ± 0.09	3.84 ± 0.05	3.32 ± 0.05	3.65 ± 0.06	3.17 ± 0.10
	> 8 Patients	250	3.35 ± 0.02	3.61 ± 0.03	3.47 ± 0.04	3.51 ± 0.04	2.73 ± 0.06	3.56 ± 0.04	3.26 ± 0.04	3.43 ± 0.04	3.22 ± 0.06
	*p*-value		< 0.001^*^	< 0.001^*^	< 0.001^*^	< 0.001^*^	0.14	< 0.001^*^	0.37	0.01^*^	0.64
Certification evaluation of dental institution^†^	Certification	108	3.39 ± 0.04	3.77 ± 0.05	3.52 ± 0.06	3.61 ± 0.06	2.55 ± 0.10	3.66 ± 0.06	3.52 ± 0.06	3.67 ± 0.07	2.86 ± 0.09
	Non certification	269	3.39 ± 0.02	3.67 ± 0.03	3.56 ± 0.04	3.57 ± 0.04	2.73 ± 0.05	3.65 ± 0.04	3.18 ± 0.03	3.44 ± 0.04	3.35 ± 0.06
	*p*-value		0.97	0.10	0.68	0.553	0.09	0.98	< 0.001^*^	< 0.001^*^	0.001^*^

By ^†^Independent t-test, ^‡^One-way ANOVA, ^a,b,c,d^Scheffe analysis, ^*^ *p* < 0.05

A: Patient safety policy across hospital units, B: Feedback and openness of communication for patient safety, C: Supervisor /manager democratic expectation/actions, D: Frequency of events reported, E: Teamwork within units for patient safety, F: System and procedure for patient safety, G: Strict manager response to error, H: Concern for error

Regarding number of hours worked per week, statistically significant differences were found in patient safety policy across hospital units, frequency of reported incidences, systems and procedures for patient safety, and concerns about errors (*p* < 0.05). Regarding facility type, significant differences were found in frequency of reported incidences, strict manager responses to errors, and concerns about errors (*p* < 0.05). Statistically significant differences were found in five areas, in addition to frequency of reported incidences and concerns about errors, with respect to the number of patients treated per day (*p* < 0.05). Significant differences between those with and those without facility certification were found regarding systems and procedures for patient safety, strict manager responses to errors, and concerns about errors (*p* < 0.05). Importantly, statistically significant differences were found for all aspects of patient safety culture by the size of the facility (*p* < 0.05).

## Discussion

This study targeted dental hygienists to examine the relationship between their workplace conditions and their workplace patient safety culture. The proportion of positive responses regarding patient safety culture was 50.4%, which was higher than 44.9% reported by a previous study on dental facility professionals and students in 2014 [30]. However, the percentage was 58% for Korean hospitals [31], which was lower than over 60% reported for the United States [32] and Taiwan [33] and indicates the relatively low patient safety culture among dental professionals.

Some aspects of patient safety culture, such as patient safety policy across hospital units, within-unit teamwork for patient safety, and supervisor/manager democratic expectation/actions, were relatively positive evaluations. Of those, within-unit teamwork for patient safety was previously found to have the highest percentage of positive responses [30–33], and it was relatively high in our study (64%). This finding indicates the extent of positive teamwork and collaboration engaged in by hygienists, but problems were implied by the small percentage (33.2%) that positively responded that there was an adequate workforce to perform all of the job requirements.

Aspects of patient safety culture with relatively low positive responses were systems and procedures for patient safety, concerns about errors, and frequency of reported incidences, with frequency of reported incidences the lowest among them (21.6%). Similar previous studies on domestic and foreign medical facilities found comparatively stronger patient safety cultures (67–68%) [31, 32], as did previous studies on dental medical facilities in the United States (47%) [34]. We could not ascertain whether the respondents’ lack of reported incidences was because there were none to report or because of other problems. Respondents at clinic-level facilities (2.81 ± 0.06) were more likely than those at hospital-level facilities (2.51 ± 0.07) to indicate reported incidences. The Patient Safety Act in Korea [4] states that safety accidents at hospitals with more than 200 beds and at general hospitals with more than 100 beds are self-reported, and hospital staffs might tend to avoid disclosing information about accidents at their hospitals [35]. Therefore, the information reported in this study is somewhat dubious.

This study’s results further found that workplace environments closely related to patient safety culture. Previous studies on hospitals found positive influences of facility size and work experience and negative influences of numbers of hours worked per week on patient safety culture [31, 36]. Our study found that patient safety culture was strongest among respondents whose numbers of hours worked per week and numbers of patients were lowest, on average, and statistically significant differences were found by type of facility. Regarding the number of hours worked per week, all of the components of patient safety culture, except systems and procedures for patient safety, were higher among those who worked less than 40 h per week. The negative influences of long work hours previously found on job performance might be a somewhat obvious link.

Regarding facility type, more respondents at clinic-level facilities than at hospital-level facilities indicated a higher patient safety culture score on five aspects. This finding supports previous results that smaller medical facilities have stronger patient safety cultures than larger medical facilities. These results might be explained by El-Jardali’s idea [37] that smaller facilities are more likely than larger ones to enjoy shared cultures, homogenous values among workers, and influential leadership. Further, the hygienists in this study who treated less than eight patients per day were more likely than those who treated eight or more patients per day to indicate a patient safety culture in six areas. Because the number of patients treated per day might negatively affect a dental facility’s patient safety culture, standards for appropriate numbers of patients must be established.

To establish high-quality care and patient safety protocols, patient safety cultures need to be established by deploying financial and institutional supports in workplace environments. However, laws and policies on patient safety in South Korea and in the Korea Institute for Dental Healthcare Accreditation do not specifically mandate dental professionals’ workplace environments for improving patient safety. Therefore, practical policies must be implemented, particularly those that ensure high quality workplace environments, such as sufficient staffing, appropriate work hours, and adequate rest, before a patient safety culture can be established.

This study selected a sample using a convenience sampling method and targeted dental hygienists in a metropolitan area, and, consequently, the results are not generalizable to all dental hygienists in South Korea. Moreover, not all of the characteristics of a patient safety culture could be analyzed because items with low reliability were necessarily excluded. Instruments appropriate for analyzing dental facilities’ patient safety cultures are lacking because most dental treatment is outpatient. Most surveys have focused on medical surgical care, and we were compelled to use a questionnaire developed for hospitals’ inpatient safety cultures. Thus, the results might not be accurate if there maybe differences between hospitals and dental facilities in terms of managing these facilities depending on their size as well as some workplace factors related to patient safety cultures. It is necessary to develop instruments specifically designed to assess dental professionals’ patient safety cultures that consider management factors and size.

Few previous studies to date have investigated the patient safety cultures of dental facilities. The current study investigated variation in that culture and the influences of various factors on it, such as the workplace environment of dental facilities. This study is meaningful because its results might help develop safer dental treatment environments by improving workplace environments.

## Conclusion

In sum, dental hygienists’ workplace environments closely relate to patient safety. Ensuring high quality workplace environments through sufficient staffing, appropriate numbers of work hours, and adequate rest must be established before a strong patient safety culture can develop.
